# The Reinforcing Effect of Waste Polyester Fiber on Recycled Polyethylene

**DOI:** 10.3390/polym14153109

**Published:** 2022-07-30

**Authors:** Jian Su, Zhiwei Jiang, Changqing Fang, Mannan Yang, Linlin Wu, Zhigang Huang

**Affiliations:** 1 Faculty of Printing, Packaging Engineering and Digital Media Technology, Xi’an University of Technology, Xi’an 710054, China; sujian@xaut.edu.cn (J.S.); jiangzhiwei0556@163.com (Z.J.); ymannan@163.com (M.Y.); 18829027972@163.com (L.W.); 2 School of Mechanical and Precision Instrument Engineering, Xi’an University of Technology, Xi’an 710048, China; 3 Key Laboratory of Processing and Quality Evaluation Technology of Green Plastics of China National Light Industry Council, Beijing Technology and Business University, Beijing 100048, China

**Keywords:** waste polyester fiber, recycled polyethylene, reinforce, composites

## Abstract

To improve the performance and application value of recycled plastics, filling modification has been widely used in waste plastic reinforcement. In this study, recycled polyethylene (RPE) was reinforced via extrusion blending using waste polyester fiber (WPF) from a waste silk wadding quilt as a reinforcer. The effects of the amount of WPF on the mechanical properties, the thermal stability of RPE and the microstructure of the RPE/WPF composite were studied. The result shows that extrusion blending can evenly disperse WPF in RPE matrix and that WPF can clearly improve the tensile strength, flexural modulus, storage modulus and thermal stability of RPE. The tensile strength and flexural modulus almost achieved the maximum when the addition of WPF was 20 wt%. The storage modulus under this condition is also higher than that of other samples. This study provides a cheap and effective reinforcement method for waste plastics as well as a new idea for the reuse of WPF, which is of great significance to the reuse of waste and environmental protection. However, how to enhance the interface adhesion between WPF and RPE to further improve the enhancement effect needs further research.

## 1. Introduction

Polyethylene is one of the most produced varieties of general-purpose synthetic resin and is widely used in industry, agriculture, packaging and daily life because of its good performance properties such as being tasteless and nontoxic, requiring low water absorption and having good chemical stability and easy molding. Consequently, the amount of waste polyethylene produced every year is also very large. Melt regeneration is currently the most environmentally friendly way to treat waste plastics, which effectively reduces the pollution and fossil energy consumption [1]. However, due to the complicated influence factor in the regeneration process, the recycled plastics often have many impurities and manufacturing defects. In addition, re-processing will also cause aging, and its performance will decline further. Therefore, recycled plastic products usually have poor quality. These problems bring great restrictions on the use of recycled plastic products. As a result, the recycled plastic materials usually need modification or enhancement.

It was found that fiber reinforcement can significantly improve the mechanical properties of polymer matrix. To improve the mechanical, thermal and other properties of polymers, different types of fibers have been tried. In recent years, the commonly used fibers for the enhancement of polymer have included glass, carbon, aramid, natural plant, nylon and polyester fibers [2,3,4]. Dun et al. [5] improved the tensile strength and impact toughness of linear low-density polyethylene/bamboo fibers composites by reinforcing the flexible interfacial binding force. Torres et al. [6] studied the interfacial properties of natural fiber-reinforced polyethylene and found that fiber treatment with stearic acid increased the interfacial shear strength by 23% with respect to untreated sisal fibers. Research shows that filling fiber materials is one of the effective ways to improve the properties of polyethylene. Polyester fiber has a high melting point and modulus, and it is one of the most produced synthetic fibers in the world. It is widely used in the production of clothing, home textile products, etc. Polyester fiber has the advantages of low cost, easy processing, easy blending with natural fibers such as cotton, high breaking strength and modulus of elasticity, good heat and light resistance, etc. With a total production of more than 50 million tons in 2016, it is growing much faster than any other synthetic fiber [7]. Like polyolefin materials, polyesters are also difficult to degrade in nature [8]. Therefore, the reuse of these waste fibers and converting them into usable products are of great importance for resource conservation and environmental protection. Polyester fiber is substantially better than polyethylene in terms of mechanical properties and thermal stability and is a good reinforcing material in theory. However, few studies focus on the reinforcement of thermoplastic plastic using waste polyester fibers. Most of the research focuses on their application for fabric polymer reinforcement, asphalt modification, concrete reinforcement, etc. [9,10].

Zarei et al. [11] used polyester fibers and calcium lignosulfonate to improve the fracture performance of asphalt concretes under freeze–thaw damage conditions. The result shows that the fracture energy and fracture toughness of the sample containing polyester fibers and calcium lignosulfonate increased compared with the base sample. Zhang et al. [12] studied the low-temperature performance of steel slag/polyester fiber permeable asphalt and the modification mechanism. Sharma et al. [13] studied the mechanical properties of cotton-based composite fabric reinforced with waste polyester fibers. Kamble et al. [14] reinforced the thermoset epoxy resin using cotton and polyester fibers that were recovered from pre-consumer textile waste. The mechanical properties of both cotton/epoxy and polyester/epoxy composites improve with fiber volume fraction. Erturk et al. [15] studied the abrasive wear performance of multilayer interwoven polyester fiber and polytetrafluoroethylene particle-reinforced polyester composite. Xu et al. [16] studied the mechanical performance of polyester fiber and SBR latex compound-modified cement concrete road overlay material for the purpose of effectively dealing with the brittleness and inferior dynamics performance of cement concrete overlay in pavement. He et al. [17] prepared novel composites with multilayer directional structure using different orientations polyester fibers to reinforce ultrahigh molecular weight polyethylene. The results show that polyester fibers can improve the ultimate flexural strength and reduce friction coefficient of the composites. It follows that waste polyester fiber is a good reinforcing material and that its application in waste plastic strengthening is worth exploring. In this study, polyester fiber fillers from a waste silk wadding quilt were used as reinforcing materials to prepare RPE/WPF composites via the most commonly used twin screw extrusion blending process. This study provides a new mechanism for the performance improvement of recycled polyolefin as well as the recycling of waste polyester fiber. The obvious strengthening effect of waste polyester fiber for thermoplastic materials was confirmed, and further study such as improving the interface adhesion and preparation technology will be carried out.

## 2. Materials and Methods

### 2.1. Materials

The recycled polyethylene was purchased from Haise Plastic Material Co., Ltd., and its pellets were produced by extruder and pelletizer from the waste polyethylene. The density and melt flow index (MFI) of the purchased recycled polyethylene were 0.929 g/cm^3^ and 3.64 ± 0.1 g/10 min (190 °C, 5 kg), respectively. The waste polyester fiber was obtained from the fiber fill of a waste silk wadding quilt.

### 2.2. Preparation of RPE/WPF Composites

The WPF was washed with deionized water to remove impurities. These long and bent fibers are intertwined with each other. To disperse the fibers and make them disperse evenly in the polyethylene matrix, the fiber mass was first kneaded into strips with a diameter of about 10 mm and then cut at intervals of about 6 mm along the length; the length of the cut short fiber is about 6–15 mm. The total mass of RPE and WPF for extrusion blending was 1000 g and the mass ratios of WPF were 0 wt%, 5 wt%, 10 wt%, 15 wt%, 20 wt% and 25 wt%, respectively.

RPE and WPF were blended by a co-rotating intermeshing twin screw extruder (SHJ-35, Nanjing Hanyi Mechanical & Electrical Co., Ltd., Nanjing, China) equipped with two parallel screws. The diameter of each screw was 35.6 mm, and the length–diameter ratio was 50. The temperatures of each heating area were 165 °C, 170 °C, 175 °C, 175 °C, 178 °C, 178 °C and 180 °C, and the temperature of the extrusion die mouth was 185 °C. The feed speed was about 10 kg/h, and the screw speed was 25 rpm. The twin screw extruder was preheated for 1 h at the preset temperature before feeding. The artificial premixed RPE and WPF mixture was poured into the feed port and extruded into strips, and then the strips were cut into particles using a granulator and dried at 100 °C for 1 h to obtain RPE/WPF composites.

### 2.3. Characterization

Type A multipurpose test specimens as specified in ISO 3167 were prepared for the tensile strength, elongation at breaking and flexural modulus tests using a horizontal injection molding machine (TY-7003, Jiangsu Tianyuan Testing Equipment Co., Ltd., Yangzhou, China). A universal testing machine (XWW-20A, Shanghai Jingjun Instruments Co., Ltd., Shanghai, China) was used to test the mechanical properties according to ISO 527-2 and ISO 178 international standards. The results were the averages of six tests and the standard deviations. The tensile strength test was carried out under the condition of a clamping distance of 115 mm and a tensile speed of 10 mm/min. The flexural modulus test was carried out under the condition of a pressing speed of 2 mm/min and a specimen span of 64 mm using three-point bending. The microstructure analysis was carried out using a field emission scanning electron microscope (SEM, JSM-6007F, JEOL, Japan) with an accelerating voltage of 20 kV. The samples were brittle fractured by freezing with liquid nitrogen and were treated with gold spraying. The thermal property of the composites was tested using differential scanning calorimetry (DSC200F3, NETZSCH-Gerätebau GmbH, Germany) with a heating rate of 10 °C/min from 25 °C to 300 °C. About 10 mg of the samples were used for testing, and the whole test process was carried out under a nitrogen atmosphere (30 mL/min). The thermal stability of the composites was analyzed with a thermogravimetric analyzer (TG209F3, NETZSCH-Gerätebau GmbH, Germany) at a heating rate of 10 °C/min from 25 °C to 600 °C. About 10 mg of the samples were used for testing, and the whole test process was carried out under a nitrogen atmosphere (50 mL/min). To study the dynamic mechanical properties of the RPE/WPF composites, dynamic mechanical analysis (DMA, METTLER TOLEDO DMA 1 STARe) in the tensile model with 1 Hz was carried out. The temperature range for the DMA measurement was between −100 °C and 150 °C with a heating rate of 5 °C/min. The specimens for DMA testing were prepared using a plate vulcanizing machine (FR-1418, Shanghai Farui Instrument Equipment Co., Ltd., Shanghai, China). The RPE/WPF composites were hot pressed into thin sheets with a thickness of about 0.5 mm with the plate vulcanizing machine and cut into a rectangular piece with a size of about 10 × 6 mm.

## 3. Results and Discussion

### 3.1. Mechanical Properties Analysis

**Figure 1:**
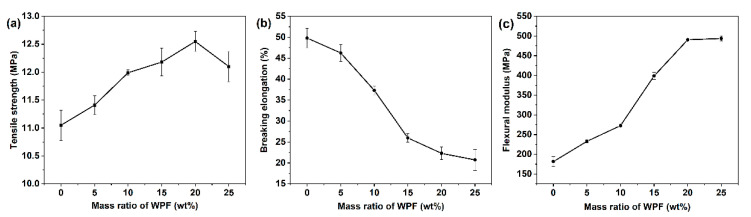
The mechanical properties of RPE/WPF composites with different WPF content: (**a**) tensile strength, (**b**) elongation at breaking, (**c**) flexural modulus.

Figure 1 shows the effects of the different WPF contents on the tensile strength (Figure 1a), elongation at breaking (Figure 1b) and flexural modulus (Figure 1c) of the RPE/WPF composites. The result shows that the tensile strength and flexural modulus of the RPE/WPF composites is obviously higher than that of RPE. However, the elongation at breaking of RPE/WPF composites decreases clearly with increasing WPF content.

Even if the WPF content is only 5 wt%, the tensile strength increases by 7.10% compared with that of RPE. When the WPF content is 20 wt%, the tensile strength of the composite reaches the maximum value of 12.55 MPa, which is 13.56% higher than that of RPE (11.05 MPa), but when the WPF content is more than 20 wt%, the tensile strength does not improve further with the increase of WPF content; instead, it goes down. From the overall trend, with the increase of WPF content, the tensile strength first increases when the WPF content is below 20 wt% and then decreases. This is because when the composites are subjected to tensile force, the tensile force can transfer to the WPF through the interface between RPE and WPF, and the WPF bears part of the tensile force, thus increasing the tensile strength. However, too many fibers can destroy the structure of the polymer itself and are more likely to cause structural defects in RPE/WPF composites, resulting in the fragmentation of the polymer matrix and decline in its tensile strength. Therefore, too much filling of WPF is not conducive to the enhancement of RPE. 

Figure 1b shows that the elongation at breaking of the RPE/WPF composites decreases gradually with the increase of WPF content. The elongation at breaking of polyester material is less than that of RPE, and the filling of WPF limits the movement ability of polymer chain, even causing stress concentration and thus reducing the elongation at breaking of the RPE/WPF composites. As the fiber has a large length-to-diameter ratio, it is easy to be oriented in the flow of plastic melt, and it has a clamping effect on the orientation of the polymer molecular chain. Therefore, the orientation of the molecular chain cannot be recovered, thus reducing the elongation at break of the prepared composites. In addition, the addition of WPF filler easily causes internal defects of the material such as cracks, voids, gaps, and impurities. When stressed, the stress in the local range near the defects increases sharply, forming stress concentration and reducing the elongation at breaking.

Figure 1c shows that with increasing WPF content (≤20 wt%), the flexural modulus of the composite increases obviously and then tends to be constant. When the WPF content is 20 wt%, the flexural modulus (490.4 Mpa) is 169.89% higher than that of RPE (181.7 Mpa). When the WPF content is 25 wt%, the flexural modulus reaches the maximum value of 493.8 Mpa, and the increase is not obvious relative to the RPE/WPF composites with 20 wt% WPF.

Overall, a proper amount of WPF (≤20 wt%) can obviously improve the tensile strength and anti-deformation capacity of RPE, but too much WPF filling will lead to declines in the mechanical properties.

### 3.2. Morphology of Fracture Surface

**Figure 2:**
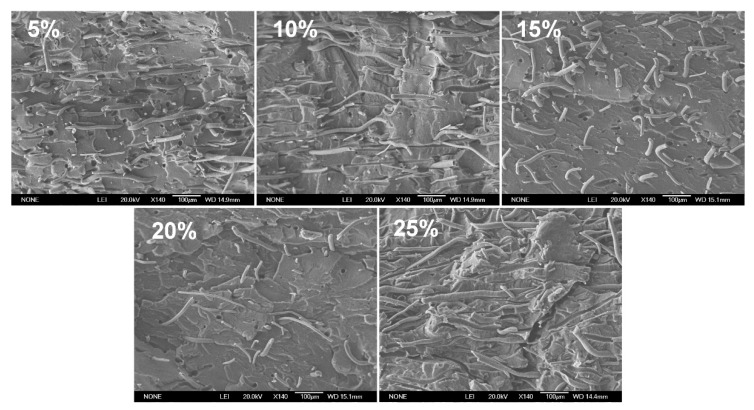
The SEM images of the fracture surfaces of RPE/WPF composites with different content of WPF (wt%).

Figure 2 shows the fracture surface morphology of the prepared RPE/WPF composites. It can be seen from Figure 2 that the WPF is uniformly dispersed in the RPE matrix and that no agglomeration or entanglement is observed. There are abundant WPF and voids exposed in the fracture surface that formed after the fibers pulled out. In addition, the surfaces of these exposed voids and WPF are relatively smooth, indicating that the interfacial adhesion between WPF and RPE matrix is weak, which greatly reduces its enhancement effect. This is because the surface of the WPF is relatively smooth itself, and it was not pretreated. Moreover, the extrusion blending temperature cannot make the WPF deform or melt, so the mechanical interlocking force is limited.

It is worth noting that the fiber distribution in the RPE matrix has obvious directivity. In the view of the fracture surface, some WPF is parallel to the fracture surface (5 wt%, 10 wt% and 25 wt%), and some is perpendicular to the fracture surface (15 wt% and 20 wt%). This is because the directional flow of the RPE/WPF composites melts during injection molding, and the fibers go in the direction of extrusion. The sides of the fracture sample were selected randomly for the morphology characterization, so the fibers in the SEM images disperse in different directions. The directional distribution of the filled WPF can cause anisotropy in the mechanical properties of the RPE/WPF composite.

### 3.3. DSC Analysis

**Figure 3:**
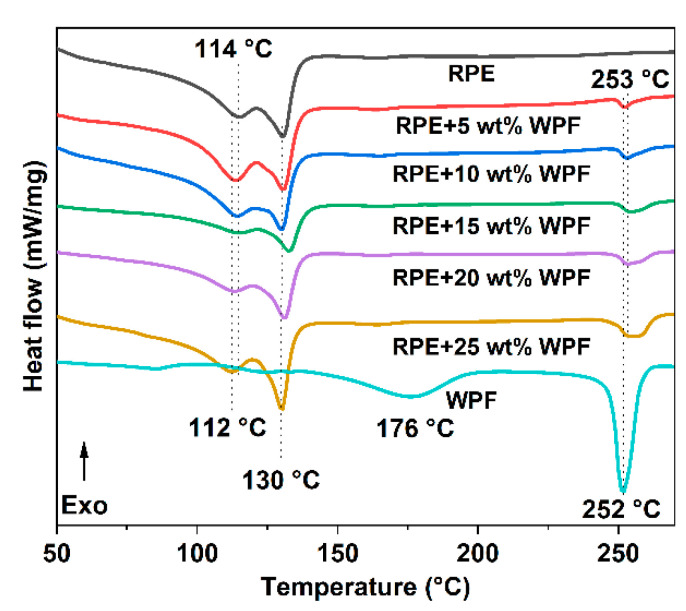
DSC curves of RPE, WPF and RPE/WPF composites with different WPF contents.

Figure 3 shows the DSC curves of RPE, WPF, and RPE/WPF composites with different WPF contents. The RPE has two obvious melting peaks, at about 114 °C and 130 °C. This could be because the purchased recycled polyethylene was derived from different types of waste polyethylene production and contained both high-density and low-density polyethylene: The melting point of low-density polyethylene is 114 °C, and that of high-density polyethylene is 130 °C. WPF shows a distinct melting peak at about 252 °C and another weak endothermic peak at about 176 °C, which is related to the heat history in the processing and molding of the polyester fiber. Polyester crystallizes slowly, and different thermal histories can result in different crystallization and melting processes. It is also possible that two different steady-state crystals are formed during crystallization and that those with poor thermal stability melt at a lower temperature whereas those with high thermal stability melt at a higher temperature. In addition, the melting point of low-density polyethylene component in the composite decreased by about 2 °C to about 112 °C because the addition of WPF affected the crystallization of low-density components, but the high-density components were less affected. The melting point of WPF in the composites increased by about 1 to 253 °C because the WPF dispersed in RPE and the heat had to be transmitted through the polyethylene matrix; thus, there is a certain hysteresis in temperature. In conclusion, since the mixture of the WPF and RPE is a physical process, no new substance is formed, and thus, its thermal properties have no obvious change.

### 3.4. Thermogravimetric Analysis

**Figure 4:**
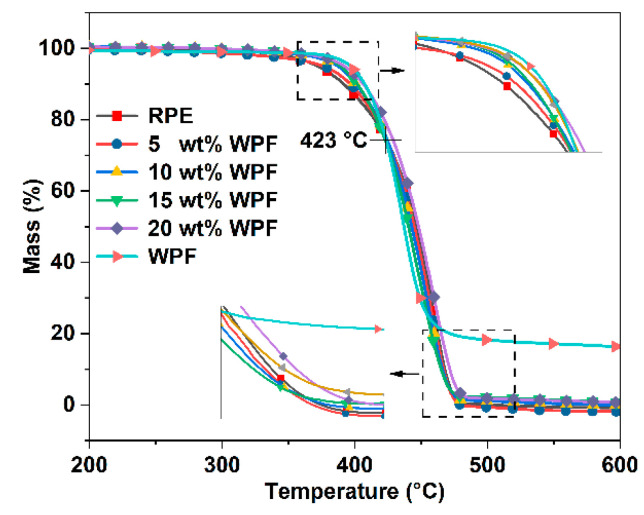
TG curves of RPE, WPF and RPE/WPF composites with different WPF contents.

Figure 4 shows the TG curves of RPE, WPF, and RPE/WPF composites with different WPF contents. Figure 4 shows that weight loss of 5 wt% corresponds to the temperatures 372 °C and 397 °C for RPE and WPF, respectively. Weight loss of 10 wt% corresponds to the temperatures 392 and 407 °C for RPE and WPF, respectively. This indicates that WPF has better thermal stability than RPE under a daily use environment. However, weight loss of 50 wt% corresponds to the temperatures 447 °C and 437 °C for RPE and WPF, respectively. It indicates that WPE has a faster decomposition rate than RPE at high temperature. The result shows that WPF has a higher initial pyrolysis temperature than RPE, but the decomposition speed of WPF is faster than that of RPE when the temperature exceeds about 423 °C. Compared with RPE, the initial cracking temperature of the RPE/WPF composites increases in varying magnitude, and the decomposition rate slows down when the temperature exceeds 423 °C. In general, the thermal stability of the RPE/WPF composites is slightly improved. In addition, almost no residue remains after RPE decomposition at 600 °C, while WPF has about 16 wt% residue. Therefore, different proportions of residue are left from the RPE/WPF composites after RPE decomposition.

### 3.5. DMA Analysis

**Figure 5:**
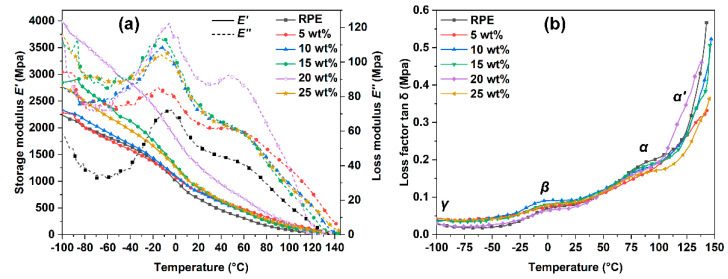
Storage modulus *E′* (**a**), loss modulus *E’’* (**a**) and loss factor tan *δ* (**b**) of RPE and RPE/WPF composites.

Figure 5 shows the dynamic mechanical properties of RPE and RPE/WPF composites. Figure 5a gives the variation trend of the energy storage modulus (*E’*) at different temperatures, which indicates the energy stored by elastic deformation during material deformation. As can be seen from the curves, with the increase of temperature, the storage modulus decreases continuously until it drops to about 0 Mpa at about 130 °C. According to the DSC data, the RPE used in this study contains both low-density polyethylene and high-density polyethylene, and the melting points are 112–114 °C and 130 °C degrees, respectively. When the temperature exceeds 130 °C, the RPE matrix is in a viscous state and completely loses its elastomeric properties and the energy storage modulus. In addition, when the WPF content is 20 wt%, the storage modulus at all temperatures is higher than all other samples. When the WPF content is less than 20 wt%, the storage modulus increases with the increase in WPF content. However, when the WPF content is over 20 wt%, the storage modulus decreases obviously with the increase of WPF content. The results are consistent with the results of the static tensile test. An appropriate content of WPF can obviously enhance the mechanical properties of RPE. However, too much filler will destroy the structure of the original polymer matrix, and it is easy to produce internal structural defects in the process of molding, resulting in stress concentration and thus reducing the performance of the composite. 

Figure 5a also gives the variation trend of the loss modulus (*E’’*) at different temperatures, which indicates the energy lost in form of heat during deformation. The peak loss modulus can reflect the viscosity of polymer melt to some extent. After adding WPF, the viscosity of the composites increases.

Figure 5b shows the loss factor tan *δ* at different temperatures. In the loss factor–temperature curves, three relaxation transition zones can be observed with the increase of temperature, which correspond to *γ*, *β* and *α* relaxation of polyethylene. Polyethylene is a partially crystalline polymer. The *γ* relaxation is at about −100 °C, which is due to the molecular chain crankshaft movement in the amorphous region and the molecular chain torsion movement in the crystalline region. The *β* relaxation is located between −70 °C and 10 °C, which is in the glassy transition region of the amorphous region and is caused by the movement of branching points. In general, low-density polyethylene has three relaxations (*γ*, *β* and *α*), while high-density polyethylene does not have *β* relaxation, and its *α* relaxation splits into two relaxes, *α* and *α’*. The samples prepared in this study show four relaxations (*γ*, *β*, *α* and *α’*), confirming that the purchased RPE contains both low-density polyethylene and high-density polyethylene, which is consistent with the DSC results. The *α* relaxation is composed of two relaxation processes with different activation energies and is caused by molecular motion in the crystalline region. The *α’* relaxation is caused by the slip of the crystal sheet [18].

## 4. Conclusions

WPF from the filling fiber of a waste silk wadding quilt was used as reinforcer to strengthen RPE. The results show that the tensile strength, flexural modulus and storage modulus of the RPE/WPF composite are clearly enhanced by WPF, while the elongation at breaking decreases to a certain extent. When the WPF content is more than 20 wt%, the increase of WPF content cannot further improve the tensile strength, flexural modulus and storage modulus of the composites. Filling with too much WPF can cause mechanical properties to deteriorate, but 20 wt% is a reasonable content. Additionally, WPF can be evenly dispersed in the RPE matrix by extrusion blending, but the interface adhesion between them is poor. The melting point and thermal stability of the RPE/WPF composites are slightly better than RPE. In conclusion, WPF is an excellent reinforcer for thermoplastic polymers that can obviously improve the mechanical properties and thermal stability of waste polymers, and it has almost no cost in raw materials. However, it is worthy of further study to improve the enhancement effect by increasing the interfacial binding force of WPF and RPE while reducing the fiber filling amount.

## Data Availability

The authors declare data availability.
